# Identifying requirements for RSK2 specific inhibitors

**DOI:** 10.1080/14756366.2021.1957862

**Published:** 2021-08-05

**Authors:** Eric B. Wright, Shinji Fukuda, Mingzong Li, Yu Li, George A. O’Doherty, Deborah A. Lannigan

**Affiliations:** a Department of Biomedical Engineering, Vanderbilt University, Nashville, TN, USA; b Department of Pathology, Microbiology & Immunology, Vanderbilt University Medical Center, Nashville, TN, USA; c Division of Cell Growth and Tumor Regulation, Proteo-Science Center, Ehime University, Toon, Japan; d Department of Chemistry and Chemical Biology, Northeastern University, Boston, MA, USA; e Department of Cell and Developmental Biology, Vanderbilt University, Nashville, TN, USA

**Keywords:** p90 ribosomal S6 kinase, RSK, RSK2, p90RSK, SL0101

## Abstract

Identifying isoform-specific inhibitors for closely related kinase family members remains a substantial challenge. The necessity for achieving this specificity is exemplified by the RSK family, downstream effectors of ERK1/2, which have divergent physiological effects. The natural product, SL0101, a flavonoid glycoside, binds specifically to RSK1/2 through a binding pocket generated by an extensive conformational rearrangement within the RSK N-terminal kinase domain (NTKD). In modelling experiments a single amino acid that is divergent in RSK3/4 most likely prevents the required conformational rearrangement necessary for SL0101 binding. Kinetic analysis of RSK2 association with SL0101 and its derivatives identified that regions outside of the NTKD contribute to stable inhibitor binding. An analogue with an *n*-propyl-carbamate at the 4” position on the rhamnose moiety was identified that forms a highly stable inhibitor complex with RSK2 but not with RSK1. These results identify a SL0101 modification that will aid the identification of RSK2 specific inhibitors.

## 1. Introduction

Identifying inhibitors that are specific for individual members of highly related proteins remains a challenge. For example, the Serine/Threonine protein kinase RSK family have ∼90% identity in their N-terminal kinase domains (NTKD), which is the domain responsible for phosphorylation of target substrates. RSK is a downstream effector of the Ras-Raf-MEK1/2-ERK1/2 (MAP kinase), a signalling cascade that is an important oncogenic driver in various cancers and this pathway has been the subject of intensive drug development efforts^1^. Treatments for cancers with an activated MAP kinase pathway include a B-Raf and MEK1/2 inhibitor combination but unfortunately, resistance inevitably occurs via reactivation of the pathway^1^. Interestingly, through an undefined mechanism, cell lines resistant to BRAF and MEK1/2 inhibitors remain dependent on RSK^2^^,^^3^. RSK is also associated with resistance against a diverse group of chemotherapeutic agents that include platinum compounds as well as sonic hedgehog, phosphoinositide 3-kinase, and heat shock protein 90 inhibitors^4–7^. Thus, targeting RSK activity independently of its upstream activators may provide novel therapeutic approaches for treatment-induced resistance.

**Figure 1.**
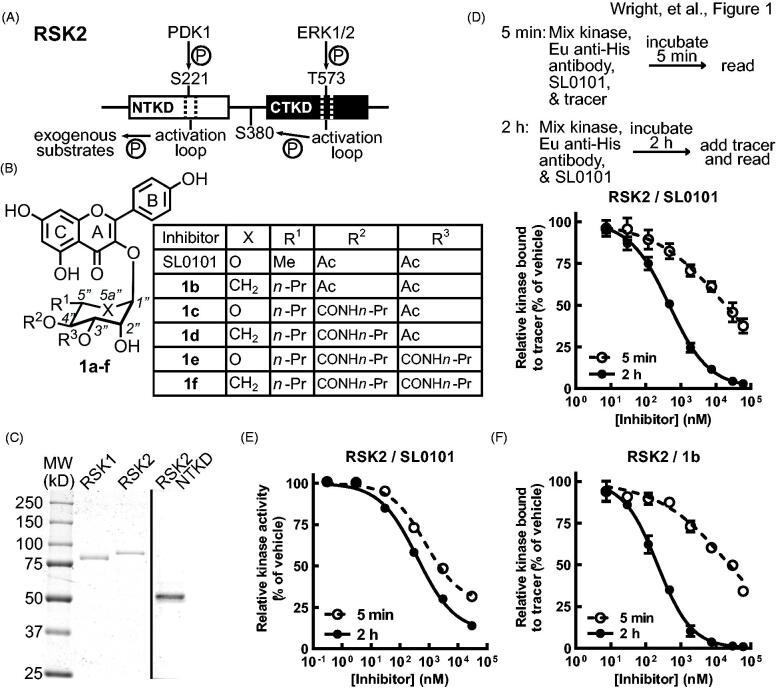
SL0101 binding to RSK2 is time dependent. (A) Simplified schematic of RSK2 kinase domains and their activation sites and functions, adapted from^39^. (B) Chemical composition of SL0101 and analogues. (C) Recombinant, purified RSK1, RSK2, and RSK2 NTKD visualised with Coomassie Blue. (D) Schematic of FRET assay (upper). Kinase, Eu-labelled anti-His antibody, SL0101, and tracer are mixed, incubated for 5 min and fluorescence measured, or a kinase/antibody/SL0101 mixture incubated for 2 h before tracer addition and fluorescence determined. Amount of tracer bound to RSK2 relative to vehicle control (lower) (*N* = 5 in quadruplicate for 5 min incubation and *n* = 9 in quadruplicate for 2 h pre-incubation). (E) Relative kinase activity in ELISA-based assay of RSK2 pre-incubated with SL0101 for 5 min or 2 h (*N* = 3 each in triplicate). (F) Relative tracer bound to RSK2 incubated with inhibitor **1b** for 5 min or 2 h (*N* = 2 in quadruplicate or 4 in quadruplicate, respectively). Data are plotted as mean and error bars represent SD.

RSK has two non-identical kinase domains, an NTKD and a C-terminal kinase domain (CTKD) (Figure 1(A)). The NTKD belongs to the protein A, G and C kinase family^8^ and the CTKD belongs to the Ca^2+^/calmodulin-dependent protein kinase II family and is necessary for autophosphorylation^8^. SL0101, a flavonoid glycoside (Figure 1(B) **1a**), was the first specific RSK inhibitor described and demonstrates specificity for inhibiting the RSK1/2 NTKD ^9–11^. The covalent inhibitors, Fmk^12^^,^^13^ and CN-NHiPR^14^, are CTKD inhibitors and are fairly specific. Dimethyl fumarate also acts as a covalent inhibitor of the RSK2 CTKD but inhibits the related MSK family^15^. The CTKD is only required for RSK activation and CTKD inhibitors do not inhibit activated RSK. Additionally, RSK activation by the CTKD can be bypassed^12^^,^^16^. Therefore, inhibition of RSK via the CTKD is context dependent, suggesting that the clinical utility of CTKD inhibitors may be limited. The pan RSK inhibitors, BI-D1870^10^^,^^17–19^ and BIX02565^20^^,^^21^, are not RSK specific and those based on the 3,4-bi-aryl pyridyl^22^ or 7-azaindole scaffold^23^ have poor pharmacokinetics. Importantly, the functions of individual RSK family members are still not clearly understood, although RSK1/2 are implicated in tumorigenesis and RSK3/4 are thought to act as tumour suppressors^5^^,^^24^.

SL0101 (**1a**) was identified during a screen of botanical extracts for RSK2 inhibition^9^. The crystal structure for the RSK2^NTKD^ in complex with SL0101 (RSK2^NTKD^-SL0101) identifies that the SL0101-binding pocket requires extensive re-arrangement of the N-lobe of the NTKD^25^. There is partial overlap between residues that comprise ATP and SL0101 binding pockets, but the sites are distinct from each other. As expected, based on the interaction mechanism a detailed analysis of the specificity of the SL0101 derivative, C5”-*n*-propyl cyclitol SL0101 (**1b**), demonstrated that **1b** primarily targeted RSK1/2 in a screen of 247 kinases, containing representatives from all kinase families^11^. Furthermore, (**1b**) was ineffective at inhibiting proliferation in a cell-based assay when its targets, RSK1/2, were silenced^26^. These data predict that SL0101-based derivatives will have limited off target effects and therefore, we have continued to evaluate and develop SL0101-based analogues.

It is now recognised that kinetic analysis of the mechanism of enzyme inhibition provides useful insights into the pharmacokinetic and pharmacodynamic properties of compounds, which can be used as a guide for further drug development^27^. Therefore, in this study we analysed the binding kinetics of RSK1 and RSK2 with SL0101 and selected derivatives. RSK2 shows time dependent SL0101 binding and the SL0101-binding pocket is likely formed by random conformational sampling by the NTKD. In contrast RSK1 does not exhibit time dependent binding to SL0101. SL0101 analogues were identified that inhibited RSK2 but not RSK1 kinase activity *in vitro* for > 12 h. Because of the extremely slow off rate of these SL0101 analogues with RSK2 we speculate that in vivo RSK2 will be the primary target of inhibition in vivo and that the biological half-life of the derivatives will be less critical for effective *in vivo* inhibition. Despite the high degree of homology between RSK1 and RSK2 our data provide a solution to the identification of RSK2 specific inhibitors. RSK2-specific inhibitors would allow the functions of RSK2 kinase activity to be identified without contributions of the kinase activity of the other RSK isoforms or the ability of RSK2 to act as a scaffold to complicate the analysis.

## 2. Materials and methods

### 2.1. Inhibitors

SL0101 was purchased from Cayman Chemical Company. Inhibitors **1b**, **1d**, and **1f** and pyran **8** were synthesised and characterised as previously described^26^^,^^28^^,^^29^. Synthesis of inhibitors **1c** and **1e** are detailed below, starting from pyran **8** (Figure S1).

#### 2.1.1. *(2 s,3R,6S)-6-((5,7-bis(benzyloxy)-2–(4-(benzyloxy)phenyl)-4-oxo-4H-chromen-3-yl)oxy)-2-propyl-3,6-dihydro-2H-pyran-3-yl* n*-propylcarbamate* (9)

To a solution of alcohol compound **8** (45 mg, 0.0646 mmol) in 0.13 ml acetonitrile, 1,8-diazabicyclo(5.4.0)undec-7-ene (0.8 μL, 6 μmol), triethylamine(46 μL, 0.33 mmol) and *n*-propyl isocyanate (20 μL, 0.2 mmol) were added successively. The resulting mixture was stirred at room temperature for 10 h. The reaction mixture was then washed with saturated sodium bicarbonate and brine, dried over magnesium sulphate, then concentrated on rotary evaporator. The resulting crude was purified by chromatography and flashed with solvent (hexanes: ethyl acetate = 4:1) to afford product as pale yellow solid, compound **9** (31 mg, 62%); R*_f_* = 0.57 (hexanes: ethyl acetate = 1:1); m.p. 120 − 122 °C; [α]_D_^20^: −136.2 (DCM, c = 2.45); ^1^H NMR (400 MHz, CDCl_3_) δ 8.00 (d, *J* = 8.7 Hz, 2H), 7.54 (d, *J* = 7.4 Hz, 2H), 7.49 − 7.31 (m, 12H), 7.29 (d, *J* = 7.3 Hz, 1H), 7.07 (d, *J* = 8.9 Hz, 2H), 6.55 (d, *J* = 1.9 Hz, 1H), 6.42 (d, *J* = 1.9 Hz, 1H), 6.23 (d, *J* = 10.2 Hz, 1H), 5.95 (d, *J* = 10.1 Hz, 1H), 5.84 (s, 1H), 5.26 (s, 2H), 5.13 (s, 2H), 5.06 (s, 2H), 4.95 (d, *J* = 9.2 Hz, 1H), 4.69 (t, *J* = 5.6 Hz, 1H), 3.46 − 3.36 (m, 1H), 3.14 (dt, *J* = 12.8, 6.5 Hz, 2H), 1.52 (m, 2H), 1.21 − 1.08 (m, 1H), 1.08 − 0.97 (m, 1H), 0.92 (t, *J* = 7.4 Hz, 3H), 0.62 (t, *J* = 8 Hz, 3H), 0.53 − 0.57 (m, 1H); ^13 ^C NMR (100 MHz, CDCl_3_) δ 173.78, 162.93, 160.45, 159.97, 159.03, 155.94, 153.85, 138.49, 136.67, 136.64, 135.89, 131.17, 130.87, 128.97, 128.91, 128.83, 128.64, 128.39, 127.88, 127.84, 127.63, 127.22, 126.85, 124.17, 114.73, 110.22, 98.36, 95.28, 94.07, 77.59, 77.47, 77.27, 76.95, 70.91, 70.66, 70.30, 70.25, 69.56, 43.03, 34.46, 23.40, 17.76, 14.63, 11.44; HRMS (MALDI-TOF/CCA): Calcd. [C_48_H_48_NO_9_ + Na]^+^: 782.3324, Found: 782.3353; IR (thin film, cm^−1^) 2965, 1724, 1606, 1508, 1365, 1251, 1226, 1200, 963, 880, 787, 739, 493, 445, 396.

#### 2.1.2. *(2 s,3R,4S,5R,6S)-6-((5,7-bis(benzyloxy)-2–(4-(benzyloxy)phenyl)-4-oxo-4H-chromen-3-yl)oxy)-4,5-dihydroxy-2-propyltetrahydro-2H-pyran-3-yl* n*-propylcarbamate* (10)

Acetate alkene **9** (65 mg, 0.083 mmol) was dissolved in 0.17 ml of a mixture of acetone and *t*-butyl alcohol (1:1), then osmium tetroxide (0.27 mg, 1.3 mol%) and 50% *N*-methylmorpholine *N*-oxide water solution (1.7 μL, 0.083 mmol) were added dropwise successively at 0 °C. The resulting mixture was stirred at room temperature overnight. After completion, the reaction mixture was washed with saturated sodium bicarbonate and brine, dried over magnesium sulphate, then concentrated on rotary evaporator. The resulting crude was purified by chromatography and flashed with solvent (hexanes: ethyl acetate = 1.5:1) to afford product as light yellow solid, compound **10** (60 mg, 72%); R*_f_* = 0.21 (hexanes: ethyl acetate = 1:1); m.p. 79–81 °C; [α]_D_^20^_:_ −131.4 (DCM, c = 1.92); ^1^H NMR (400 MHz, CDCl3) δ 7.85 (d, *J* = 8.7 Hz, 2H), 7.52 (d, *J* = 7.4 Hz, 2H), 7.47 − 7.30 (m, 12H), 7.28 (d, *J* = 7.3 Hz, 1H), 7.07 (d, *J* = 8.8 Hz, 2H), 6.53 (d, *J* = 1.7 Hz, 1H), 6.42 (s, 1H), 5.69 (s, 1H), 5.25 (s, 2H), 5.13 (s, 2H), 5.05 (s, 2H), 4.81 (t, *J* = 5.8 Hz, 1H), 4.62 (t, *J* = 9.2 Hz, 1H), 4.42 (s, 1H), 4.01 –-3.90 (m, 1H), 3.82 (d, *J* = 5.1 Hz, 1H), 3.55 (s, 1H), 3.27 (t, *J* = 10.3 Hz, 1H), 3.09 (dd, *J* = 13.3, 6.4 Hz, 2H), 1.94 (s, 1H), 1.54 − 1.35 (m, 3H), 1.28 − 1.16 (m, 2H), 1.16 − 1.02 (m, 1H), 0.87 (t, *J* = 7.0 Hz, 3H), 0.83 − 0.68 (m, 1H), 0.64 (t, J = 7.2 Hz, 3H); ^13 ^C NMR (100 MHz, CDCl3) δ 173.60, 163.07, 160.63, 160.00, 159.00, 157.46, 153.92, 137.62, 136.52, 135.81, 130.75, 128.97, 128.92, 128.83, 128.66, 128.43, 127.92, 127.81, 127.56, 126.91, 126.85, 123.60, 114.97, 110.08, 100.40, 98.44, 94.05, 74.69, 71.55, 70.95, 70.69, 70.63, 70.56, 70.35, 43.13, 33.84, 23.27, 18.26, 18.20, 14.42, 14.34, 11.40; HRMS (MALDI-TOF/CCA): Calcd. [C_48_H_49_NO_11_ + Na]^+^: 838.3198, Found: 838.3199; IR (thin film, cm^−1^) 3352, 2961, 2873, 1699, 1603, 1570, 1438, 1404, 1352, 1251, 1176, 1100, 1048, 1001, 950, 822, 735, 696, 622, 543, 465.

#### 2.1.3. *(2 s,3R,4S,5S,6S)-2-((5,7-bis(benzyloxy)-2–(4-(benzyloxy)phenyl)-4-oxo-4H-chromen-3-yl)oxy)-3-hydroxy-6-propyl-5-((propylcarbamoyl)oxy)tetrahydro-2H-pyran-4-yl acetate* (11)

To a solution of compound diol **10** (18.7 mg, 0.023 mmol) in 0.23 ml acetonitrile, 2-aminoethyl diphenylborinate (0.5 mg, 10 mol%), propyl isocyanate (2.5 μL, 0.035 mmol) and DIPEA (8 μL, 0.046 mmol) were added successively. The resulting mixture was stirred at room temperature for 12 h. Reaction mixture was then washed with saturated sodium bicarbonate and brine, dried over magnesium sulphate, then concentrated on rotary evaporator. The resulting crude was purified by HPLC and flashed with solvent (hexanes: ethyl acetate = 1.5:1) to afford product as yellow oil, compound **11** (12 mg, 57%): R*_f_* = 0.32 (hexanes: ethyl acetate = 1:2); m.p. 115 − 117 °C; [α]_D_^20^: −130.0, (c = 1.1, DCM); ^1^H NMR (400 MHz, CDCl_3_) δ 7.88 (d, *J* = 8.6 Hz, 2H), 7.54 (d, *J* = 7.6 Hz, 2H), 7.48 − 7.33 (m, 11H), 7.30 (d, *J* = 7.3 Hz, 1H), 7.16 − 7.08 (m, 2H), 6.56 (d, *J* = 2.2 Hz, 1H), 6.45 (d, *J* = 2.2 Hz, 1H), 5.71 (d, *J* = 2.3 Hz, 1H), 5.38 − 5.28 (m, 1H), 5.25 (s, 2H), 5.15 (s, 2H), 5.11 (d, *J* = 7.5 Hz, 1H), 5.07 (s, 2H), 4.89 (t, *J* = 9.4 Hz, 1H), 4.62 (t, *J* = 6.0 Hz, 1H), 4.52 (s, 1H), 3.23 (m, *J* = 9.2, 7.0 Hz, 1H), 3.11 (dt, *J* = 13.6, 6.9 Hz, 1H), 3.00 (dt, *J* = 13.3, 6.5 Hz, 1H), 2.80 (s, 1H), 2.09 (s, 3H), 1.44 (dt, *J* = 14.5, 7.3 Hz, 2H), 1.37 − 1.15 (m, 3H), 1.09 (dt, *J* = 13.6, 7.2 Hz, 1H), 0.87 (t, *J* = 7.4 Hz, 3H), 0.73 (m, *J* = 7.0 Hz, 1H), 0.64 (t, *J* = 7.1 Hz, 3H). ^13 ^C NMR (100 MHz, CDCl_3_) δ 11.3, 14.3, 14.4, 18.1, 21.3, 23.4, 33.8, 42.9, 69.7, 70.4, 70.7, 71.0, 71.7, 72.2, 94.1, 98.5, 100.5, 110.1, 115.1, 123.6, 126.9, 127.6, 127.9, 128.0, 128.4, 128.7, 128.9, 128.9, 129.0, 130.8, 135.8, 136.5, 136.6, 137.5, 154.1, 155.7, 159.1, 160.0, 160.7, 163.1, 170.4, 173.5.; HRMS (MALDI-TOF/CCA): Calcd. [C_50_H_51_NO_12_ + Na]^+^: 880.3303, Found: 880.3315; IR (thin film, cm^−1^) 3434, 2962, 2930, 2871, 1726, 1573, 1486, 1454, 1437, 1353, 129, 1249, 1176, 1103, 1047, 1002, 954, 806, 736, 697.

#### 2.1.4. *(2 s,3R,4S,5S,6S)-2-((5,7-dihydroxy-2–(4-hydroxyphenyl)-4-oxo-4H-chromen-3-yl)oxy)-3-hydroxy-6-propyl-5-((propylcarbamoyl)oxy)tetrahydro-2H-pyran-4-yl acetate* (1c)

To a solution of compound **11** (4.6 mg, 0.005 mmol) in 0.1 ml methanol, Pd/C (0.1 mg, 10 mol%) was added and resulting mixture was stirred under a H_2_ atmosphere for 24 h. The reaction mixture was loaded onto silica gel and eluted with hexane-EtOAc (1:2) to give inhibitor **1c** (2.3 mg, 72%); R_*f*_ = 0.78 (EtOAc); m.p. 110 − 112 °C; [α]_D_^19^: −73.0 (MeOH, c = 0.3); ^1^H NMR (400 MHz, CD_3_OD) δ 7.85 (d,  = 8.2 Hz, 2H), 6.95 (d, *J* = 8.5 Hz, 2H), 6.38 (d, *J* = 2.3 Hz, 1H), 6.20 (d, *J* = 2.3 Hz, 1H), 5.74 (s, 1H), 5.26 − 5.12 (m, 1H), 4.95 − 4.90 (m, 2H), 4.34 (d, *J* = 3.1 Hz, 1H), 3.06 − 2.96 (m, 2H), 2.95 − 2.79 (m, 1H), 2.06 (s, 3H), 1.44 (td, *J* = 14.4, 7.2 Hz, 3H), 1.32 − 1.03 (m, 5H), 0.87 (m, *J* = 6.3, 5.3 Hz, 4H), 0.64 (t, *J* = 7.2 Hz, 3H); ^13 ^C NMR (100 MHz, CD_3_OD) δ 178.2, 171.0, 164.9, 162.0, 160.5, 157.3, 157.1, 157.0, 134.0, 130.8, 121.3, 115.6, 104.6, 100.5, 98.8, 93.6, 72.5, 72.1, 69.5, 68.4, 42.3, 33.4, 22.8, 19.7, 18.0, 13.1, 10.3; HRMS (MALDI-TOF/CCA): Calcd. [C_29_H_33_NO_12_ + Na]^+^:610.1895, Found: 610.1896; IR (thin film, cm^−1^) 3320, 3261, 2963, 2064, 1979, 1725, 1657, 1610, 1508, 1365, 1277, 1209, 1175, 1121, 975, 819, 657, 457.

#### 2.1.5. *(2 s,3S,4S,5R,6S)-6-((5,7-bis(benzyloxy)-2–(4-(benzyloxy)phenyl)-4-oxo-4H-chromen-3-yl)oxy)-5-hydroxy-2-propyltetrahydro-2H-pyran-3,4-diyl bis(*n*-propylcarbamate)* (12)

To a solution of compound diol **10** (35 mg, 0.046 mmol) in 0.4 ml acetonitrile, 2-aminoethyl diphenylborinate (1 mg, 10 mol%), propyl isocyanate (8.7 μL, 0.092 mmol) and triethylamine (22 μL, 0.132 mmol) were added successively. The resulting mixture was stirred at room temperature for 12 h. Then reaction mixture was washed with saturated sodium bicarbonate and brine, dried over magnesium sulphate, then concentrated on rotary evaporator. The resulting crude was purified by HPLC and flashed with solvent (hexanes: ethyl acetate = 1.5:1) to afford product as yellow oil, compound **12** (16 mg, 40%); R*_f_* = 0.34 (hexanes: ethyl acetate = 1:1.5); [α]_D_^20^: −144.9 (DCM, c = 0.73) ; ^1^H NMR (400 MHz, CDCl_3_) δ 7.88 (d, *J* = 8.5 Hz, 2H), 7.54 (d, *J* = 7.4 Hz, 2H), 7.48 − 7.26 (m, 11H), 7.13 (d, *J* = 8.6 Hz, 2H), 6.55 (d, *J* = 1.8 Hz, 1H), 6.44 (d, *J* = 1.6 Hz, 1H), 5.76 (s, 1H), 5.25 (s, 2H), 5.18 (s, 1H), 5.15 (s, 1H), 5.11 (s, 1H), 5.06 (s, 2H), 4.87 (t, *J* = 9.6 Hz, 1H), 4.67 (s, 1H), 4.53 (s, 1H), 3.17 (s, 1H), 3.10 (t, *J* = 11.0 Hz, 2H), 3.06 − 2.99 (m, 1H), 1.55 − 1.32 (m, 5H), 1.29 − 1.22 (m, 1H), 1.18 (d, *J* = 5.5 Hz, 2H), 1.08 (dd, *J* = 12.3, 6.4 Hz, 1H), 0.90 (t, *J* = 7.4 Hz, 3H), 0.84 (t, *J* = 7.4 Hz, 3H), 0.75 − 0.66 (m, 1H), 0.62 (t, *J* = 7.0 Hz, 3H); ^13 ^C NMR (100 MHz, CDCl_3_) δ 173.52, 170.35, 163.10, 160.66, 160.03, 159.05, 155.70, 154.09, 137.51, 136.57, 136.51, 135.83, 130.83, 128.99, 128.92, 128.85, 128.68, 128.43, 127.94, 127.84, 127.58, 126.91, 123.56, 115.11, 110.10, 100.52, 98.46, 94.10, 72.17, 71.68, 70.98, 70.76, 70.71, 70.37, 69.67, 42.94, 33.83, 23.37, 21.28, 18.14, 18.10, 14.43, 14.32, 11.30; HRMS (MALDI-TOF/CCA): Calcd. [C_52_H_56_N_2_O_12_ + Na]^+^: 923.3725, Found: 923.3718; IR (thin film, cm^−1^) 3587, 2963, 2872, 1718, 1605, 1509, 1377, 1251, 1176, 997, 955, 889, 802, 736, 646.

#### 2.1.6. *(2 s,3S,4S,5R,6S)-6-((5,7-dihydroxy-2–(4-hydroxyphenyl)-4-oxo-4H-chromen-3-yl)oxy)-5-hydroxy-2-propyltetrahydro-2H-pyran-3,4-diyl bis(*n*-propylcarbamate)* (1e)

To a solution of bis-carbamate compound **12** (7.5 mg, 0.008 mmol) in 0.05 ml methanol, Pd/C (0.4 mg, 10 mol%) was added and resulting mixture was stirred under a H_2_ atmosphere for 24 h. The reaction mixture was loaded onto silica gel and elution with hexane-EtOAc (1:1.5) to give inhibitor **1e** (3.5 mg, 67%); R*_f_* = 0.12 (hexanes-EtOAc = 1:2); [α]_D_^20^: −110.1 (MeOH, c = 0.15), m.p. 158 − 160 °C; ^1^H NMR (400 MHz, CD_3_OD-d4) 7.86 (d, *J* = 8.7 Hz, 2H), 6.97 (d, *J* = 8.5 Hz, 2H), 6.38 (d, *J* = 1.5 Hz, 1H), 6.20 (d, *J* = 1.6 Hz, 1H), 5.72 (s, 1H), 5.08 (dd, *J* = 10.2, 2.7 Hz, 1H), 4.35 (s, 1H), 3.08 (t, *J* = 6.8 Hz, 2H), 3.00 (t, *J* = 6.9 Hz, 2H), 1.48 (m, *J* = 29.4, 14.5, 7.2 Hz, 5H), 1.17 (m, *J* = 18.2, 16.9, 10.1 Hz, 4H), 0.93 (t, *J* = 7.4 Hz, 3H), 0.87 (t, *J* = 7.4 Hz, 4H), 0.64 (t, *J* = 7.1 Hz, 3H); ^13 ^C NMR (100 MHz, Acetone-d6) δ 178.80, 164.69, 162.84, 160.62, 157.57, 156.34, 156.29, 131.38, 122.08, 116.18, 105.32, 101.65, 99.24, 94.22, 94.16, 73.18, 72.04, 69.59, 43.01, 42.94, 33.98, 23.71, 23.48, 18.36, 14.00, 11.24, 11.13; HRMS (MALDI-TOF/CCA): Calcd. [C_31_H_38_N_2_O_12_ + Na]^+^: 653.2317, Found: 653.2314; IR (thin film, cm^−1^) 3377, 2963, 2874, 1698, 1652, 1607, 1508, 1457, 1358, 1262, 1207, 1171, 967, 949, 841, 813, 768, 580, 521.

### 2.2. Fret kinase assay

The relative amount of inhibitor bound to RSK2 was determined using the LanthaScreen Eu Kinase Binding Assay for RPS6KA3 (Invitrogen). Kinase, Eu anti-His antibody, inhibitor or vehicle, and Kinase Tracer 236 (tracer) in kinase buffer were incubated as described in Figure 1(D), with final assay concentrations of 5 nM kinase, 2 nM antibody, inhibitor or vehicle (0.95% DMSO), and 15 nM tracer in Proxiplate white 384-well plates (Perkin Elmer) with 15 μL total assay volume per well. For RSK1 and RSK2 NTKD, 2 nM Eu Streptavidin and 2 nM Biotin anti-His antibody (Invitrogen) and for RSK3/4, 2 nM Eu anti-GST antibody (Invitrogen) were used in place of the Eu anti-His antibody. Time resolved fluorescence was measured with a Synergy Neo 2 Multi Mode Plate Reader and Gen5 software (Biotek) with excitation fluorescence at 330 ± 80 nm and emission measured at 620 ± 10 nm (background fluorescence from Eu antibody) and at 665 ± 8 nm (emission from fluorescence resonance energy transfer, FRET, from tracer) with 100 μs delay and 200 μs collection time. For each time point, emission ratio (EMR) was calculated as emission at 665 nm divided by emission at 620 nm. The corresponding average EMR of wells without inhibitor or kinase was subtracted, and background-subtracted EMR was normalised to average vehicle control. Normalised EMR was calculated in MATLAB (Mathworks).

### 2.3. Protein expression and purification

The cDNA plasmids for human RSK1 and RSK2 were obtained from Addgene (RSK1: #70574; RSK2: #70578) as Gateway Entry Clones. Recombinant baculoviruses were generated by the Gateway cloning using the BaculoDirect Baculovirus Expression System (Invitrogen) according to the manufacturer's manual.The cDNA for RSK2 NTKD was amplified by conventional PCR, then cloned into pENTR-D-TOPO (pENTR Directional TOPO Cloning Kits, Invitrogen). The resulting Gateway Entry vectors were used for making the baculoviruses in the same way as RSK1 and RSK2 WT viruses. These constructs have 6xHis sequence in their C-termini and recombinant proteins were purified by Ni-NTA Agarose (Qiagen). The molecular weight and concentration of purified proteins were confirmed by standard SDS-PAGE and CBB R-250 staining.

### 2.4. Kinase activity assay

The activity of RSK2 was measured as previously reported ^9^. Briefly, 1 nM kinase, inhibitor or vehicle (1% DMSO), and 10 μM ATP in kinase buffer were dispensed into the wells of a 96-well plate with adsorbed glutathione *S*-transferase fusion protein containing the sequence RRRLASTNDKG. The reaction was stopped after 30 min, during the linear phase, with the addition of equal volume 500 mM EDTA, then phosphorylated peptide was measured by ELISA. Luminescence from wells with EDTA added before ATP was subtracted, then luminescence was normalised to the average vehicle luminescence. For the dilution reaction, 100 nM RSK2 was incubated with 5 μM SL0101 or 1.5 μM **1c** in kinase buffer. After 2 h incubation, the kinase/inhibitor mix was diluted 100-fold in kinase buffer. As the control reaction, 1 nM RSK2 was incubated with 0.05 μM SL0101 or 0.015 μM **1c** for 2 h. After the dilution, kinase/inhibitor solution and 3.3 μM ATP in kinase buffer were mixed to initiate the reaction. The reaction was stopped at the time indicated in the figures with the addition of EDTA, and amount of phosphorylated peptide was measured by ELISA.

### 2.5. Molecular modeling

**Figure 2.**
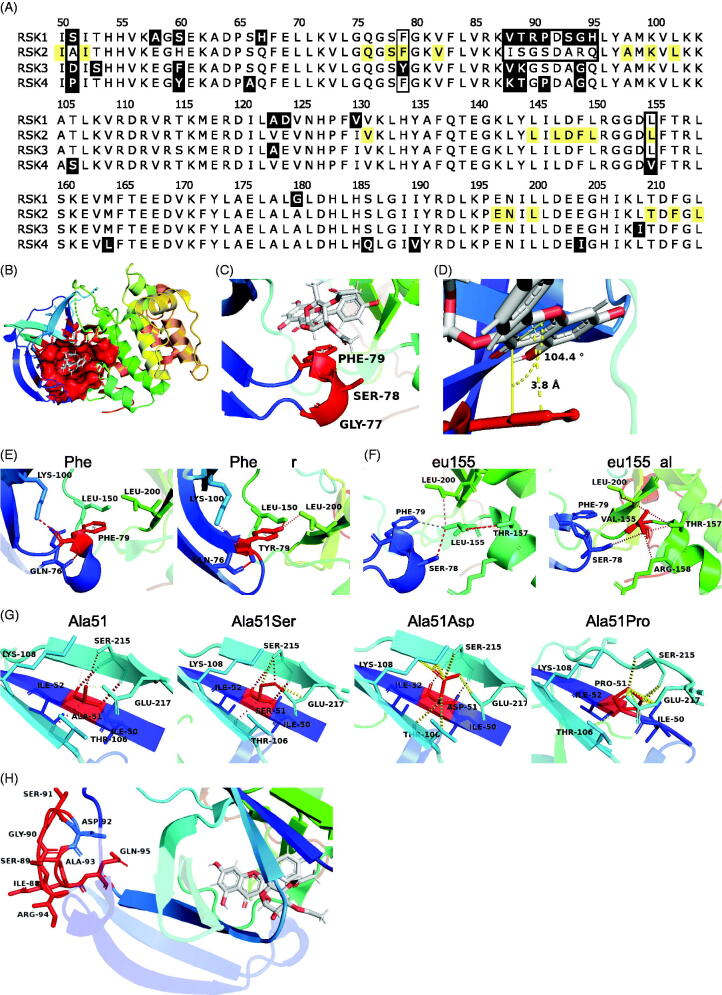
Specificity of SL0101 for RSK1/2 cannot be explained by SL0101-interacting residues. (A) Alignment of the RSK family NTKD catalytic core. Numbering and highlighting are relative to RSK2. Black = non-identical amino acids. Yellow = amino acids composing the SL0101-binding pocket. Black outline = residues discussed. (B) Crystal structure of RSK2^NTKD^-SL0101 (PDB Accession 3UBD) showing SL0101 (white) in its binding pocket (red surface). (C,D) Position of the P-loop relative to SL0101 in RSK2^NTKD^-SL0101. Distance measured between centre of Phe79 side chain phenol and A ring of SL0101, and angle measured between plane of A ring of SL0101 and Phe79. (E) Phe79 showing hydrogen bonding (red) to nearby residue side chains (left) and computational mutation of Phe79 to Tyr with additional hydrogen bonds to Leu150 and Leu200 (right). (F) Leu155 showing hydrogen bonding (red) and hydrophobic interactions (grey) to nearby residue side chains (left); computational mutation of Leu155 to Val with additional hydrogen bonds to Arg158 and Thr157 and loss of hydrophobic interaction with Phe79 (right). (G) Ala51 positioned in the middle β strand of the de novo β sheet formed in RSK2^NTKD^-SL0101 (left). Computational mutations to Ser (second to left), Asp (second to right), and to Pro (right) showing hydrogen bonding (red) or clashes between van der Waals radii (yellow). (H) Ile88 - Ser91 and Ala93 - His95 in RSK2^NTKD^-SL0101 in an unstructured region (red) clustered away from the SL0101 (white) binding pocket.

Sequences of the RSK family NTKD were aligned in Uniprot (Uniprot Consortium). Single acid mutations of the RSK2^NTKD^ in complex with SL0101 (Protein Databank Accession 3UBD) were simulated in DynaMut (University of Melbourne)^30^. Briefly, Dynamut uses normal mode analysis to predict the impact of mutations on protein conformation and stability. The Pymol (Schrodinger) session generated by Dynamut was used to visualise changes in bonding and steric interference. Dynamut removes SL0101 during modelling, resulting in some rotamer positions that would interfere with SL0101. Those rotamer positions were edited to the next lowest strain conformation that would not interfere with SL0101. The mutagenesis wizard was used to visualise steric clashes between side chains, which were then marked (Figure 2 yellow dashes). Hydrogen bonds (Figure 2 red dashes) were generated by Dynamut. To determine favorability for π-bond stacking, the distance and angle between the A ring of SL0101 and RSK2^NTKD^-SL0101 Phe79 was measured using the measurement wizard. Pseudoatoms were placed in each ring centre, then distance was measured between the pseudoatoms. Angle was measured between the plane of SL0101 A ring and the γ carbon of Phe79.

### 2.6. Quantification and statistical analysis

IC_50_ was determined by four parameter logistic curve, with top constrained to equal 100% and minimum greater than or equal to 0% but otherwise unconstrained. Hill slope and IC_50_ were unconstrained. Changes in IC_50_ over time were fit by simple linear regression during the linear portion, less than 15 min, or log-log line over longer periods. Curve fitting and statistics were calculated in Prism 9.0.2 (Graphpad). Statistical test methods are reported in the figure legends. Statistical significance is considered using α = 0.01.

## 3. Results

### 3.1. Time dependence of RSK2 binding to SL0101

To further understand the mechanism of RSK1/2 binding to SL0101 (**1a**) we expressed and purified His-tagged recombinant proteins, wild type (WT) RSK2, WT RSK1 and the isolated RSK2^NTKD^ domain, containing residues 1–389 (Figure 1(C)). Initially, a fluorescence resonance energy transfer (FRET) assay was used to measure SL0101 binding. This assay uses an ATP-mimetic tracer labelled with an acceptor fluorophore and a recombinant His-tagged kinase, which is indirectly labelled with a europium donor chelated to an anti-His antibody. Interaction of the tracer with the kinase generates a FRET signal. A tracer concentration of 15 nM was used in all FRET assays, which generated a robust signal-to-noise for all constructs (Figure S2). For IC_50_ determination the maximum signal was set to 100% as defined by the vehicle controls. The IC_50_ for the recombinant, purified RSK2 with SL0101 was ∼ 0.4 µM, which is based on a 2 h pre-incubation time of the inhibitor before tracer addition (Figure 1(D) and Table 1). GST-tagged RSK3 and RSK4 were purchased and tested with a labelled anti-GST antibody in the FRET assay. SL0101 did not inhibit RSK3 and RSK4 (Table 1), which is in agreement with the results from the ZLYTE screen we previously reported ^11^. RSK3 and RSK4 were inhibited by the pan-RSK inhibitor, BI-D1870, demonstrating that the assay was functional (Table 1). Unexpectedly, decreasing the pre-incubation time with SL0101 from 2 h to 5 min increased the IC_50_ by ∼ 40-fold for RSK2 (Figure 1(D) and Table 1). In contrast to SL0101, the efficacy of BI-D1870 to inhibit RSK2 showed no time dependence (Table 1). These data are consistent with observations showing that BI-D1870 is an ATP competitive inhibitor^31^. To determine whether the IC_50_ time dependence was unique to the FRET assay we also measured the IC_50_ in an ELISA-based assay that was used in the original screen of botanical extracts^9^. In the activity assay the kinase is pre-incubated with or without the inhibitor in solution with a substrate that is adsorbed onto a hydrophilic, protein-binding surface. The substrate contains the RSK-phosphorylation motif contained in oestrogen receptor alpha (ERα) in which Ser-167 ERα is targeted for phosphorylation^32^. ATP is used to initiate the reaction and phosphorylation is measured by an anti-phospho-Ser-167 antibody. The reaction is stopped during the linear phase. In agreement with previous reports using the ELISA assay the IC_50_ for SL0101 (**1a**) inhibition of RSK2 was ∼ 0.4 µM, which is based on a 2 h pre-incubation time of the inhibitor before initiating the kinase reaction. Pre-incubation of RSK2 with SL0101 for 5 min resulted in a more modest increase in the IC_50_ (∼ two-fold) than that obtained in the FRET assay, most likely because the kinase continues to be incubated with SL0101 over the 30 min kinase assay resulting in further inhibitor binding (Figure 1(E) and Table 1). The time dependence of RSK2 for SL0101 binding is consistent with the requirement of the extensive conformational change required to form the SL0101-binding pocket and provides support for the ability of RSK2 to form this pocket in solution^25^. Support for this hypothesis is provided by the observations that RSK2 has a higher affinity for **1b** after a 2 h incubation compared to SL0101 in both the FRET and ELISA-based assays (Figures 1(D,E) and Table 1). Inhibitor **1b** (Figure 1(B)) was designed to take advantage of a hydrophobic pocket present in the SL0101-binding pocket and therefore, its higher binding affinity than SL0101 is consistent with our assumption.

**Table 1.** Binding characteristics of SL0101 and selected analogues.

Assay	Kinase	Inhibitor	Kinase-inhibitor incubation time	IC_50_ [nM] (95% C.I.)		N	Relative Off-Rate [log_10_(nM/min)] (95% C.I.)		N	% Uninhibited kinase at 30 μM		
										Mean	±	S.D.
FRET Kinase Binding	RSK2	SL0101	2 h	423	(389 to 453)	9	0.529	(0.509 to 0.550)	7	4.3	±	1.1
			5 min	16.8 μM	(3.6 to 24.0 μM)	5				45.7	±	5.8
		**1b**	2 h	215	(189 to 236)	4	0.601	(0.564 to 0.643)	4	1.3	±	1.2
			5 min	24.1 μM	(4.2 to 30.5 μM)	2				49.7	±	1.2
		**1c**	2 h	37.4	(36.4 to 38.3)	5	0.126	(0.105 to 0.152)	5	0.3	±	0.5
			5 min	6.4 μM	(5.0 to 7.3 μM)	3				26.2	±	2.8
		**1d**	2 h	26.3	(23.7 to 29.0)	3	0.548	(0.501 to 0.591)	3	0.5	±	1.1
		**1e**	2 h	83.7	(74.1 to 91.4)	3	0.417	(0.379 to 0.461)	3	0.8	±	0.6
		**1f**	2 h	90.9	(80.5 to 102.7)	3	0.630	(0.586 to 0.674)	3	1.3	±	1.2
		BI-D1870	2 h	17.3	(16.6 to 18.1)	2				1.2	±	0.3
			5 min	11.8	(10.3 to 13.5)	2				1.1	±	0.3
	RSK2 NTKD	SL0101	2 h	426	(375 to 487)	4				11.5	±	1.7
			5 min	879	(670 to 1217)	4				20.1	±	2.4
		**1c**	2 h	12.0	(9.9 to 14.7)	2				11.1	±	1.2
	RSK1	SL0101	2 h	193	(136 to 290)	4	0.277	(0.223 to 0.333)	4	16.7	±	3.1
			5 min	774	(577 to 1104)	4				19.2	±	2.9
		**1c**	2 h	12.8	(11.7 to 14.0)	6	0.176	(0.136 to 0.224)	3	6.1	±	1.3
	RSK3	SL0101	2 h	N.D.		2				90.3	±	2.2
		BI-D1870	2 h	3.9	(2.75 to 5.09)	2				0.1	±	1.4
	RSK4	SL0101	2 h	N.D.		2				70.9	±	7.0
		BI-D1870	2 h	22.0	(15.8 to 28.3)	2				2.4	±	3.7
ELISA Kinase Activity	RSK2	SL0101	2 h	392	(290 to 555)	3				13.9	±	0.7
			5 min	739	(544 to 1057)	3				31.6	±	1.3
		**1b**	2 h	183	(126 to 274)	3				15.7	±	3.3
		**1c**	2 h	84.7	(59 to 123)	3				9.4	±	1.4
		**1d**	2 h	74.1	(53 to 107)	3				11.1	±	1.6
	RSK1	SL0101	2 h	70.7	(50 to 102)	3				3.9	±	0.6

### 3.2. Modelling of RSK2 interactions with SL0101

To gain further insight into the specificity of RSK1/2 for SL0101 we performed a pairwise alignment of RSK1, RSK3, and RSK4 NTKD to that of RSK2, and found that they have ∼ 90% identity (Figure 2(A)). The crystal structure of the RSK2^NTKD^ in complex with SL0101 (RSK2^NTKD^-SL0101) shows the hydrophobic SL0101 binding pocket comprises 22 amino acid residues, and 11 of these residues interact with SL0101 by Van der Waals force or hydrogen bonding (Figure 2(B))^25^. To explain the specificity of RSK1/2 for SL0101, single amino acid mutations were introduced computationally into the crystal structure of the RSK2^NTKD^-SL0101 complex representing amino acids from RSK1/3/4. In the RSK family, the SL0101-interacting amino acids are identical in RSK1 and differ by one amino acid each in RSK3 and RSK4 compared to RSK2 at Phe79 and Leu155, respectively. Phe79 lies at the tip of the P-loop comprising Gly77, Ser78, and Phe79 at the end of two β strands in RSK2^NTKD^-SL0101 (Figure 2(C)). In RSK2^NTKD^-SL0101, the tip of the P-loop is positioned such that the aglycone A ring of SL0101 and the aromatic ring of Phe79 lie 3.8 Å apart and 14.9° above parallel, a conformation favourable for π-bond stacking (Figure 2(D)). In this position, the aromatic ring of a Tyr residue, as in RSK3, could participate in π-bond stacking, and the *ortho*-hydroxyl could form additional hydrogen bonds with the side chains of Leu150 and Leu200 on the other side of the SL0101-binding pocket, and therefore, the Tyr residue seems unlikely to interfere with binding of SL0101 (Figure 2(E)). Leu155 lies at the end of an α helix and forms a hydrogen bond with the B ring of SL0101 (Figure 2(F)). Substitution of Leu155 with a Val residue, as in RSK4, results in additional weak hydrogen bonds with Thr147 and Arg158 and loses hydrophobic interaction with Phe79. However, these changes appear unlikely to result in destabilisation of the α helix or loss of interaction with SL0101. Taken together, it is unlikely that differences between RSK2 and RSK3/4 at Phe79 and Leu155 result in the observed differences in specificity for SL0101.

In addition to differences within residues that directly interact with SL0101 in RSK2 we investigated contributions of residues outside of the SL0101-binding pocket. These residues are mostly located in unstructured or undefined regions in the RSK^NTKD^-SL0101 crystal structure or would appear to not result in changes in charge or hydrophilicity. However, Ala51 is of interest as it is located the middle β strand (β1’) of a β sheet that uniquely forms in RSK2^NTKD^-SL0101, and its identity differs between all four RSK isoforms (Figure 2(G)). Substitution of Ala51 with a Ser residue, as in RSK1, adds a hydrogen bond with the nearby Glu217 side chain, but also sterically interferes with the Ser215 side chain on the adjacent β strand but neither of these changes appear likely to destabilise the β sheet. In contrast, an Asp residue in place of Ala51, as in RSK3, is sterically strained with the side chains of Thr106, Lys108, Ser215, and Glu217. Substitution of Ala51 with a Pro residue, as in RSK4, destabilises the entire *de novo* β-sheet by loss of main chain interactions with Ser215 on the adjacent β strand and steric interference with the side chains of Ser215 and Glu217. Previously, we found that mutation of either Ile50 or Ile52, the SL0101-interacting residues adjacent to Ala51, resulted in loss of interaction with either the nucleotide analogue AMP-PNP or SL0101, suggesting the formation of the *de novo* β sheet is necessary for the formation and stability of the SL0101-binding pocket^25^. Based on these observations we propose that Ala51 is important in the stability of the *de novo* β sheet formed in the RSK2^NTKD^-SL0101 complex and thus the replacement of this residue as occurs in RSK3/4 accounts for their inability to interact with SL0101.

### 3.3. SL0101 binding is consistent with conformational sampling

**Figure 3.**
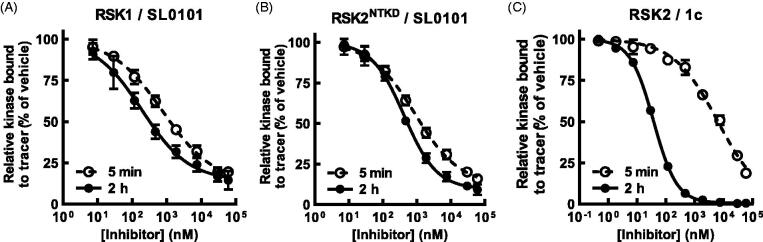
SL0101 binds differentially to RSK1 and isolated RSK^NTKD^ compared to RSK2. (A,B) Amount of tracer bound to RSK1 or RSK2^NTKD^ after 5 min or 2 h incubation with SL0101 relative to vehicle control (*N* = 4 in quadruplicate each). (C) Amount of tracer bound to isolated RSK2 after 5 min or 2 h incubation with inhibitor **1c** relative to vehicle control (*N* = 5 and 3, respectively, each in quadruplicate). Data are plotted as mean and error bars represent SD.

To investigate whether the time dependent binding of SL0101 was unique to RSK2 the interaction of SL0101 with RSK1 was investigated. In the FRET assay the affinity of RSK1 for SL0101 is ∼ two-fold higher than RSK2 (Figure 3(A) and Table 1). However, in contrast to RSK2 the length of pre-incubation of RSK1 with SL0101 did not substantially alter the IC_50_. Incubation of RSK1 with SL0101 for 2 h compared to 5 min had only a three-fold higher affinity compared to the > 40-fold difference observed with RSK2. Furthermore, a 5 min incubation with SL0101 is sufficient for 80% inhibition of RSK1 in contrast to RSK2 where only ∼ 50% inhibition occurs (compare Figures 1(D,A), Table 1). These data suggest that RSK1 is predominately in the conformation that facilitates the interaction with SL0101 compared to RSK2. In support of this suggestion RSK1 differs from RSK2 in an unstructured region comprising 7 non-identical amino acids (Figure 2(H)) and although this region does not interact with SL0101, it undergoes significant conformational rearrangement upon SL0101 binding the RSK2^NTKD^^25^. Therefore, changes in flexibility could profoundly affect the formation and stability of the SL0101-binding pocket; although, the current state of protein modelling is unable to assess such a claim.

We also analysed the binding kinetics of the isolated NTKD (RSK2^NTKD^) and observed in the FRET-based assay that the IC_50_ of RSK2^NTKD^ for SL0101 with a 2 h pre-incubation was similar to that obtained for WT RSK2 (Figure 3(B) and Table 1). But like RSK1, the IC_50_ of RSK2^NTKD^ for SL0101 increased by only ∼ two-fold and ∼ 80% inhibition achieved with a 5 min incubation, and we conclude from these data that domains outside of the NTKD inhibit the formation of the SL0101-binding pocket in RSK2.

The formation of the SL0101-binding pocket in RSK2 could occur by an induced fit mechanism or alternatively the pocket could be created during spontaneous movements that occur in the kinase independent of the presence of SL0101. In the induced fit model, we predict that there would be an initial interaction of SL0101 with the NTKD most likely through the ATP-binding pocket to generate a low affinity interaction (Figure S3A). This low affinity binding could induce a conformational change to generate the high affinity site. In the spontaneous model SL0101 would bind after conformational sampling by the NTKD generated the SL0101-binding pocket (Figure S3B). Support for the pocket occurring by conformational sampling is provided by observations with **1c** (Figure 1(B)). Inhibitor **1c** achieves the lowest IC_50_ at 5 min pre-incubation among all the analogues evaluated (Figure 3(C) and Table 1). However, an 8-fold higher concentration than the IC_50_, which should result in occupancy of the low affinity sites, results in only 75% SL0101 inhibition. It would be expected in the induced fit model that high concentrations of SL0101 should completely occupy the low affinity sites resulting in 100% inhibition. The inability to obtain complete inhibition with saturating concentrations of inhibitor is consistent with a model in which the formation of the SL0101-binding pocket occurs by conformational sampling.

### 3.4. SL0101 analogues interact with regions outside the SL0101-binding pocket

**Figure 4.**
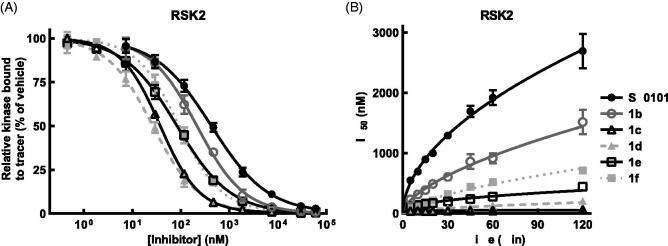
SL0101 analogues have improved affinity for RSK2 and dissociate more slowly. (A) Amount of tracer bound to RSK2 after 2 h pre-incubation with SL0101 analogues (*N* = at least 3 each in quadruplicate); Data are plotted as mean and error bars represent SD. (B) Relative off-rate of SL0101 analogues (*N* = at least 3 each in quadruplicate). Data are plotted as IC_50_ and error bars represent SE of the IC_50_. Change in IC_50_ over time is plotted as log-log linear.

In previous SAR analysis we found that addition of the *n*-propyl at the 5” position in combination with an *n*-propyl-carbamate at the 4” position (**1d**) or with *n*-propyl-carbamate groups at both the 3” and 4” positions (**1f**) (Figure 1(B)) improved the affinity of RSK2 for these analogues, which were confirmed in this study using the FRET assay^28^ (Figures 4(A) and Table 1). These results were unexpected because in the crystal structure of the RSK2^NTKD^ in complex with SL0101 (RSK2^NTKD^-SL0101) the acetyl groups of SL0101 interact with the solvent^25^; however, it is possible that these residues might be available to interact with other regions of the polypeptide in the full length kinase. This hypothesis is supported by our observations that acetylation of the hydroxy groups on the rhamnose increased the affinity of RSK2 for this analogue, which would not be expected to improve interactions with the solvent because of their hydrophobic nature^33^. In compounds **1d** and **1f** the ring oxygen in the rhamnose was replaced with a methylene group to create a cyclitol as this change could improve in vivo stability by removing electron density in the *O*-glycosidic bond^34^. For this study compounds **1c** and **1e** (Figure 1(B)) were synthesised to investigate whether the ring oxygen influenced the *in vitro* affinity, but the IC_50_s were similar to the cyclitol versions (Figures 3(C),4(A) and Table 1).

### 3.5. SL0101 analogues show differential interaction with RSK2 compared to RSK1

**Figure 5.**
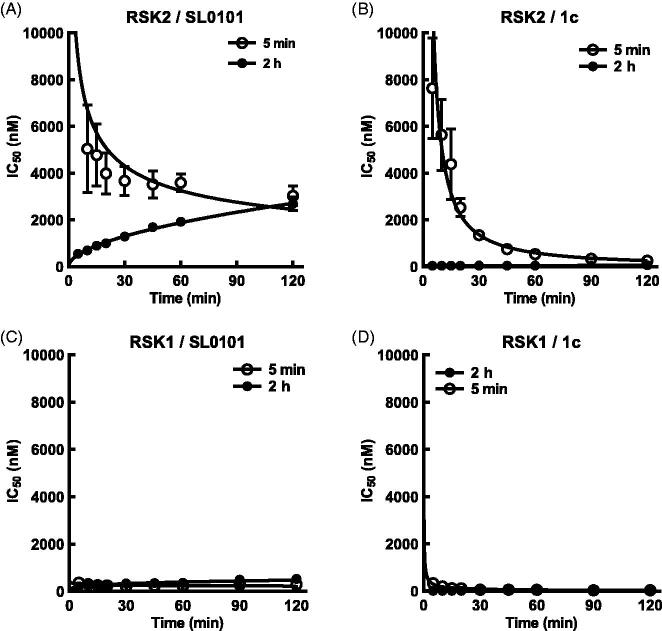
SL0101 and analogues interact differentially with RSK2 compared to RSK1. (A) Relative off-rate (change in IC_50_ after 2 h pre-incubation, *N* = 6 in quadruplicate) and on-rate (change in IC_50_ after 5 min incubation, *N* = 3 in quadruplicate) between SL0101 and RSK2, (B) inhibitor **1c** and RSK2 (*N* = 5 and 2, respectively, each in quadruplicate), (C) SL0101 and RSK1 (*N* = 4 in quadruplicate each), or (D) inhibitor **1c** and RSK1 (*N* = 4 and 2, respectively, each in quadruplicate). Data are plotted as IC_50_ with error bars representing SE of the IC_50_. Change in IC_50_ over time is fit as log-log-linear.

To further examine the interaction of SL0101 and its analogues with RSK2 and RSK1 we added tracer simultaneously with inhibitor or after a 2 h pre-incubation with inhibitor and monitored the IC_50_ over time. The results from the pre-incubation experiments provide an indirect measure of the inhibitor off rate whereas the simultaneous addition of inhibitor and tracer provide an indirect measure of the inhibitor on rate. Measuring the slope of the rate of IC_50_ change for both the pre-incubation and simultaneous experiments shows that the off rate of RSK2 for SL0101 is greater than the on rate, with a ratio of the absolute value of the off/on equal to ∼ 1.3 (Figures 4(B),5(A) and Tables 1, 2). This same analysis for inhibitor **1c** shows the inhibitor on rate for RSK2 is greater than the inhibitor off rate, with an off/on ratio equal to ∼ 0.1 (Figures 4(B), 5(B) and Table 2). The off rate for **1c** was more than three-fold lower than that of **1d**, **1e,** and **1f**, which is not reflective of their relative IC_50_s (Figures 3(C), 4(A) and Table 1). The mechanism that accounts for the unique properties of RSK2 interacting with **1c** are unknown. RSK1 has a higher off than on rate for SL0101, with an off/on ratio of ∼2.7, suggesting that the RSK1 complex with SL0101 is less stable than that of RSK2 (Figure 5(C) and Table 2). RSK1 also has a higher off/on ratio for **1c** than does RSK2, suggesting that the RSK1 complex is less stable than the RSK2 complex with **1c** (Figure 5(D) and Table 2).

**Table 2 Kinetic parameters of SL0101 and 1c.** 

Inhibitor	Kinase	Relative off-rate ([log_10_(nM/min)]	Relative on-rate [log_10_(nM/min)]	Off: On Ratio
SL0101	RSK2	0.529	−0.407	1.30
	RSK1	0.277	−0.102	2.71
**1c**	RSK2	0.126	−1.204	0.10
	RSK1	0.176	−0.719	0.25

**Figure 6.**
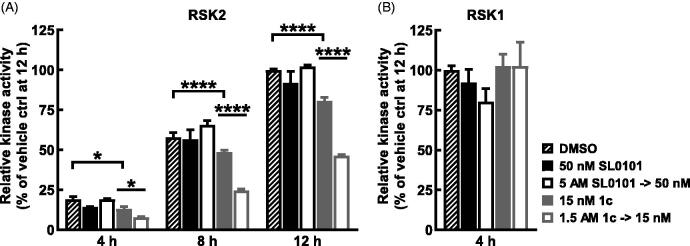
Long term inhibition of RSK2 compared to RSK1 with inhibitor **1c**. (A,B) RSK2 or RSK1 were pre-incubated 2 h with SL0101, inhibitor **1c**, or vehicle at the assay concentration shown (control) or 100X assay concentration and then diluted with kinase buffer (dilution). After dilution, ATP was added and the reaction proceeded for the times indicated, then the reaction was stopped with EDTA and the amount of phosphorylated substrate was measured by ELISA. RSK2 activity is relative to vehicle control at 12 h and relative RSK1 activity is relative to vehicle at 4 h. (*N* = 2 in duplicate each, 2-way ANOVA with Fisher’s Uncorrected LSD for multiple comparisons between control and dilution for each timepoint, *p*-values: * < 0.01, **** < 10^−5^). Data are plotted as mean and error bars represent SD.

To further analyse the stability of RSK2 with **1c** the ELISA assay was used as an orthogonal approach. For these experiments an inhibitor concentration ∼ 15-fold above the respective IC_50_ obtained in the ELISA assay was incubated with RSK2 for 2 h and then diluted 100-fold. Inhibition of kinase activity was measured at differing time points and compared to that obtained with a concentration of inhibitor equal to the concentration after dilution. SL0101 was unable to inhibit RSK2 at 4 h after the dilution whereas inhibitor **1c** maintained ∼50% inhibition over a 12 h time course (Figures 6(A)). In agreement with the analysis of the stability of RSK1 with **1c** RSK1 was not inhibited by **1c** at 4 h after the dilution (Figure 6(B)) in the ELISA assay. These results confirm the FRET results that RSK2 and inhibitor **1c** form a stable complex.

## 4. Discussion

Members of the RSK family share high homology, which has made it difficult to identify isoform specific RSK inhibitors. In support of this statement SL0101 is currently the only identified RSK inhibitor that specifically targets RSK1/2 but not RSK3/4. In this report using modelling studies we identified a key residue outside of the SL0101-binding pocket that appears to account for the specificity of RSK1/2 compared to RSK3/4. We also identified an analogue of SL0101 with 4” *n*-propyl-carbamate substitutions on the rhamnose that forms a highly stable complex with RSK2 but not RSK1, which raises the possibility of developing a novel series of compounds that would result in preferential inhibition of RSK2 *in vivo*. Unfortunately, these analogues were ineffective at inhibiting RSK in cell-based assays and we propose that it is likely due to the inability of the compounds to enter the cell.

The conformational change required to generate the SL0101-binding pocket in RSK2 is constrained by regions outside the NTKD in comparison to RSK1. This hypothesis is based on the observations that RSK1 and the RSK2^NTKD^ do not show a time dependence for binding to SL0101 and its analogues as observed with WT RSK2. The physiologic relevance for the greater flexibility of RSK1 to form the SL0101-binding pocket compared to RSK2 is not known. However, unlike RSK1, RSK2 is able to translocate to the nucleus and most likely requires other protein factors to aid in this translocation^35^. It is possible that the conformational change that generates the SL0101-binding pocket regulates the interaction with other proteins and that control of the stability of this conformation is important in regulating the functions of RSK1 versus those of RSK2.

The residence time the inhibitor interacts with the target has been shown to be a better predictor of *in vivo* efficacy than affinity^27^^,^^36^^,^^37^. Thus, identifying that the off rate for RSK2 and SL0101 analogues with 4” *n*-propyl-carbamate substitutions are extremely slow suggests that pharmacokinetic properties should not be a major driver for further drug development.

## Supplementary Material

Supplemental Material
