# Supplementary figures and images for: Identifying requirements for RSK2 specific inhibitors
Source: J Enzyme Inhib Med Chem. 2021 Aug 5;36(1):1798–809. doi: 10.1080/14756366.2021.1957862 (PMC8344253; doi:10.1080/14756366.2021.1957862)

**A** *De novo* retrosynthetic approach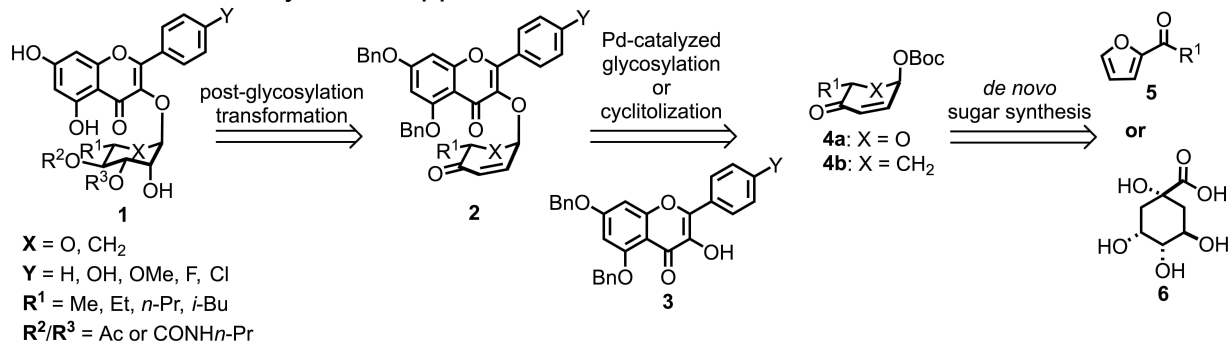**B** Synthesis of pyran **8**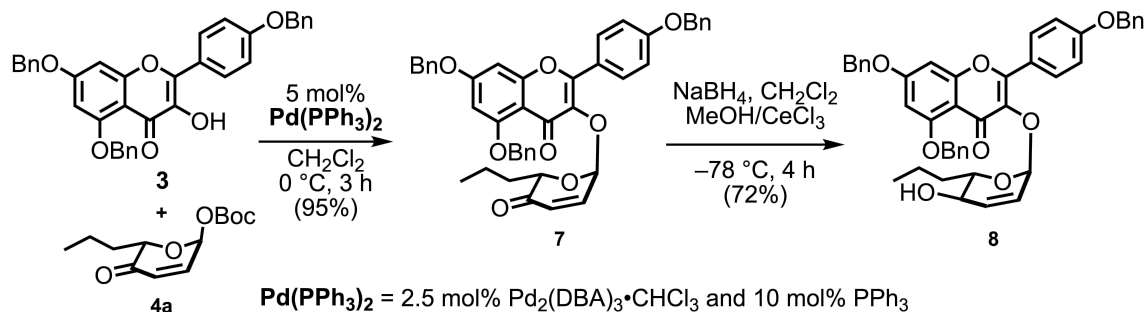**C** Synthesis of inhibitors **1c** and **1e**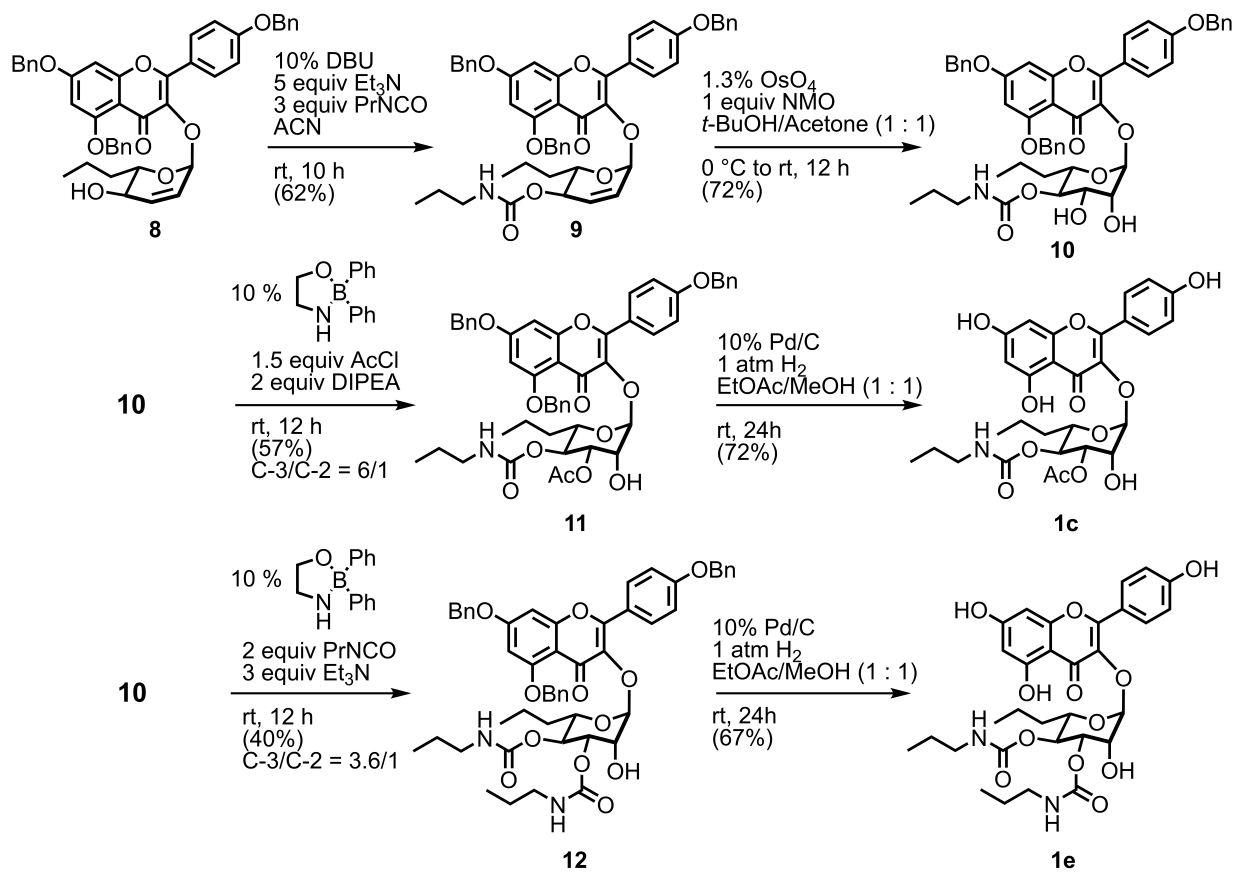

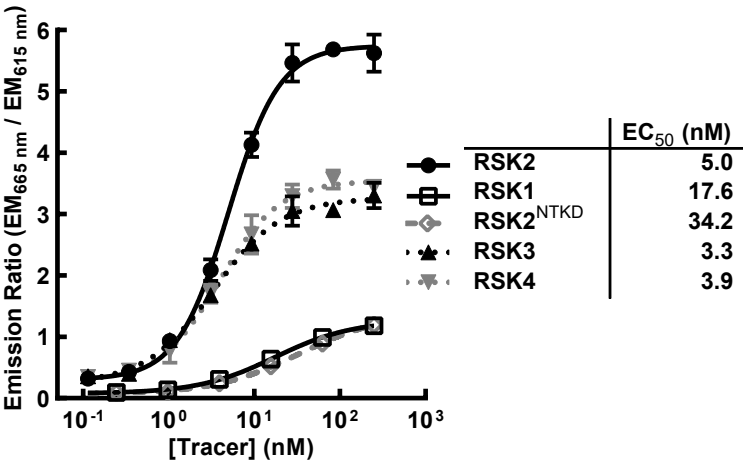

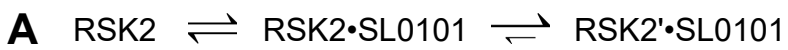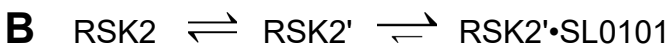

Supplement: Supplemental Material [file IENZ_A_1957862_SM6472.pdf]
